# Initial three‐ and half‐year machine performance assessment of a superficial radiation therapy unit

**DOI:** 10.1002/acm2.70258

**Published:** 2025-09-21

**Authors:** Yongsook C. Lee, William Romaguera, Stephen D. Davis, D Jay Wieczorek, Vibha Chaswal, Ranjini Tolakanahalli, Minesh P. Mehta, Noah S. Kalman, Alonso N. Gutierrez

**Affiliations:** ^1^ Department of Radiation Oncology Miami Cancer Institute Baptist Health South Florida Miami Florida USA; ^2^ Department of Radiation Oncology Herbert Wertheim College of Medicine Florida International University Miami Florida USA

**Keywords:** kilovoltage x‐ray, machine performance, quality assurance, SRT‐100 Vision, superficial radiation therapy

## Abstract

**Purpose:**

This work reports on results of periodic machine quality assurance (QA) tests for our superficial radiation therapy (SRT) unit (SRT‐100 Vision) performed for the first three‐ and half‐ year.

**Methods:**

Results of machine warm‐up, dosimetry, mechanical, safety and imaging QA tests for our SRT‐100 Vision unit were reviewed and analyzed. Dosimetry tests included output constancy, backup timer, timer accuracy, beam quality (half value layer [HVL]), applicator factors (AFs), absolute output calibration, output constancy with varying x‐ray tube head rotation, output reproducibility, output linearity, timer and end‐effect error, percent depth dose (PDD) verification and congruence between radiation field and applicator size. Mechanical tests encompassed unit stability as well as applicator integrity and indicators. Safety tests consisted of visual and audio monitors, beam‐on indicator, interlocks, and energy/filter indicators. Imaging tests for ultrasound covered functional check, spatial integrity, and image quality check. The QA tests were performed for three kilovoltage (kV) x‐ray energies (50, 70, and 100 kV_p_) and six applicators (1.5, 2.0, 2.5, 3.0, 4.0, and 5.0 cm in diameter).

**Results:**

Daily mandatory machine warm‐up was successfully completed on each day of treatment. The results of all dosimetry tests for three energies were within the recommended tolerance. Daily, and monthly outputs and annual absolute outputs were < ± 3.0%, < ± 2.0%, and < ± 1.3%, respectively. Backup timer displayed timer set ± 0.0%. Timer accuracy was < ± 1.0 sec. HVLs and AFs were < ± 1.1% and < ± 0.3%, respectively. Outputs for different head angles were < ± 1.0% and output reproducibility was within 0.1%. Output linearity was < ± 0.6% and end‐effect errors were < ± 0.0006 min. PDDs were < ± 2.2%. Congruence was < ± 0.5 mm. Monthly mechanical tests confirmed mechanical robustness of our unit. Daily safety features were verified to be functional each day. Monthly imaging tests for ultrasound showed good functionality, image quality, and spatial integrity meeting the set tolerance.

**Conclusions:**

The QA results demonstrated high performance and stability of our SRT‐100 Vision unit over the first three and a half years of operation.

## 1 INTRODUCTION

Superficial radiation therapy (SRT) is one of the treatment options for non‐melanoma skin cancer such as basal cell carcinoma and squamous cell carcinoma or for some noncancerous skin conditions, for example, keloid scars.^1^, ^2^, ^3^ While there are various SRT modalities available including low energy kilovoltage (kV) x‐rays (superficial x‐rays, ≤100 kV_p_),^4^ low energy electrons and high dose rate brachytherapy utilizing either a radioactive source or a kV source,^2^, ^5^ superficial x‐rays have been widely employed in dermatology and radiation oncology clinics due to their cost‐effectiveness and operational simplicity.^6^

**FIGURE 1:**
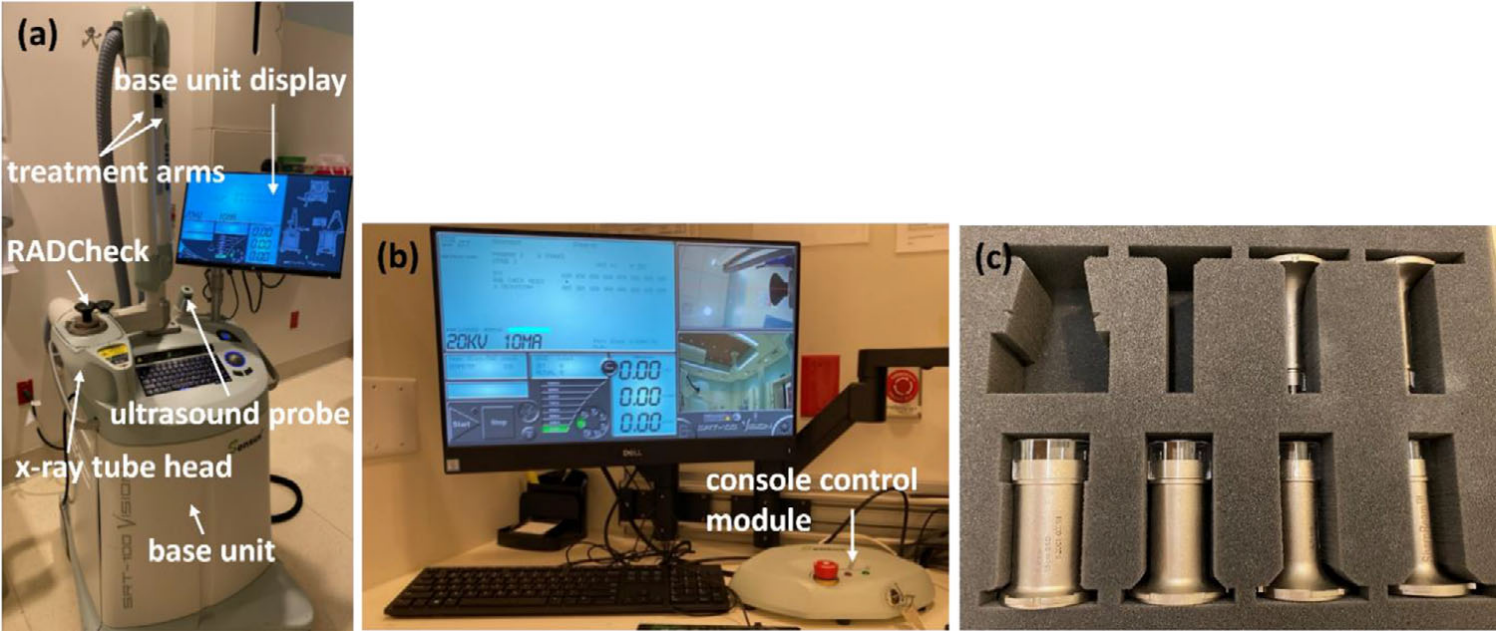
(a) Sensus Healthcare SRT‐100 Vision treatment unit with labelled components, (b) SRT‐100 operator console, and (c) SRT‐100 applicators of varying sizes.

Over several years, Sensus Healthcare (Boca Raton, FL) introduced a series of SRT units: SRT‐100, SRT‐100+, and SRT‐100 Vision.^7^ All three systems consist of a base unit housing the x‐ray apparatus, ancillary control, and service elements, as well as an operator console, and a console control module (Figure 1). The highest x‐ray energy of the systems is 100 kV_p_ and various sizes of applicators at different focal spot distances (FSDs, defined as the distance from source to applicator end) are available. The latest unit, SRT‐100 Vision, has a high frequency (20 MHz) ultrasound module used for image guided RT (Figure 1).

Following published guidelines for kV x‐ray radiotherapy machines,^8^, ^9^, ^10^ in June of 2021, an SRT‐100 Vision unit was commissioned (three energies: 50, 70, and 100 kV_p_ (Table 1); six applicators: 1.5, 2.0, 2.5, 3.0, 4.0, and 5.0 cm at FSD of 15 cm) in our clinic^7^ and periodic machine quality assurance (QA) protocols were established, incorporating dosimetry, mechanical, safety, and imaging tests. This work presents an overview of the machine QA tests implemented for our SRT‐100 Vision unit and reports on the QA results spanning three and a half years, from July 2021 to December 2024. To our knowledge, this is the first published report on long‐term performance of an SRT‐100 Vision unit.

**TABLE 1:** Three commissioned energies and their filtration of our SRT‐100 Vision unit.

Energy	50 kV_p_	70 kV_p_	100 kV_p_
Filter name	SRT50	SRT70	SRT100
Thickness and material of filter	0.45 mm Al	0.75 mm Al	1.15 mm Al

Abbreviations: Al: aluminum; kVp: peak kilovoltage; SRT: superficial radiation therapy.

## 2 METHODS

Table 2 lists dosimetry, mechanical, safety and imaging QA tests along with recommended frequency and tolerance for each QA test. Details of the QA tests are described below:

**TABLE 2:** Periodic machine quality assurance (QA) tests, frequency, and tolerance implemented for our SRT‐100 Vision unit.

Test		Frequency	Tolerance
**Warm‐up**	Machine warm‐up	Daily	Successful completion
**Dosimetry tests**	Output constancy	Daily	Baseline ± 3.0%
		Monthly	Baseline ± 3.0%
	Backup timer	Daily	Timer set ± 5.0%
	Timer accuracy	Daily	Timer set ± 1.0% or ± 1.0 sec, whichever is greater
	Beam quality: HVL_1_	Annual	Baseline ± 10.0%
	Applicator factors	Annual	Baseline ± 3.0%
	Absolute output calibration (AAPM TG‐61)	Annual	Baseline ± 3.5%^a^
	Output constancy with varying x‐ray tube head rotation	Annual	±2.0% from output with head straight
	Output reproducibility	Annual	2.0% (standard deviation/mean×100.0%)
	Output linearity	Annual	±1.0%
	Timer and end‐effect error	Annual	±0.01 min
	PDD verification: spot checks	Annual	Baseline ± 3.0%
	Congruence between radiation field and applicator size at FSD	Annual	±2.0 mm
**Mechanical tests**	Mechanical stability and safety	Monthly	Functional
	Applicator integrity and indicators	Monthly	Functional
**Safety tests**	Cameras and monitors	Daily	Functional
	Audio monitor	Daily	Functional
	Beam‐on indicator	Daily	Functional
	Interlocks (door, key, beam‐off)	Daily	Functional
	Energy/filter indicators	Daily	Functional
**Imaging tests**	Functional check	Daily	Functional
	Spatial integrity: measurement accuracy	Monthly	±1.0 mm
	Image quality: artifact	Monthly	No significant artifact

Abbreviations: AAPM, American Association of Physicists in Medicine; FSD, focal spot distance; HVL1, first half value layer; PDD, percent depth dose; SRT, superficial radiation therapy; TG, task group.

^a^ Combined uncertainties for the in‐air method of the AAPM TG‐61 protocol (AAPM TG‐61 Table III)^4^.

### 2.1 Warm‐up

#### 2.1.1 Machine warm‐up (daily)

Mandatory warm‐up of the SRT‐100 Vision unit was performed on the day of treatment. RADCheck (vendor provided diode device) was attached to the x‐ray tube head (Figure 1a). A pre‐programed warm‐up procedure ran for all available energy modes (20–100 kV_p_) with built‐in automatic filters (six in total). Upon completion of the warm‐up, an output difference (%) from the last output calibration saved in the unit was indicated on the console screen for each energy. The machine warm‐up process ensures that all energies are ready for treatment and no energy whose output exceeds 3.0% (tolerance for output recalibration set by the vendor) is confirmed.

### 2.2 Dosimetry tests

#### 2.2.1 Output constancy (daily, monthly)

**FIGURE 2:**
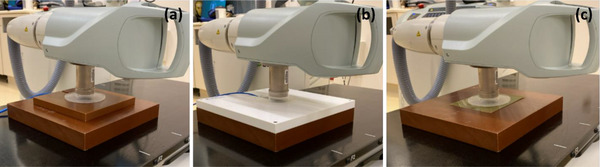
SRT‐100 setups for (a) daily output constancy check, (b) monthly output constancy check, and (c) measurement of congruence between radiation field and applicator size.

Output constancy was checked on the day of treatment at minimum for the energy utilized for treatment. Slab phantoms (size: 30.0 cm × 30.0 cm × 4.0 cm, 20.0 cm × 20.0 cm × 2.0 cm, Virtual Water, water equivalent material with a density of 1.04 g/cm^3^, CNMC, Nashville, TN),^11^ an ionization chamber (TN34045, PTW‐Freiburg, Freiburg, Germany) and a polystyrene foil (Sensus Healthcare, to remove electron contamination) were sequentially placed on a table as shown in Figure 2a. The 5.0 cm applicator was attached to the x‐ray tube head and centered on the chamber. An energy level and a corresponding filter (50 kV_p_/SRT50, 70 kV_p_/SRT70, or 100 kV_p_/SRT100) were selected and the timer was set to 0.3 min in service mode. The beam was then delivered 3–4 times with an electrometer bias set to +300 V (MAX‐4000, Standard Imaging, Inc., Middleton, WI). Charges (pC) were recorded, and a temperature and pressure correction was applied to the average charge (pC). The measured relative output was compared with the baseline established immediately following absolute output calibration. The ionization chamber, PTW TN34045, was used to avoid the use of the same ionization chamber for monthly output constancy check. The 5.0 cm applicator was selected as it is the reference applicator for absolute output calibration. The timer was set to 0.3 min for QA efficiency after output linearity for timer settings from 0.1 min to 1.0 min was confirmed. The daily output constancy check ensures that output for the energy used on the day of treatment is consistent to deliver the intended radiation dose to patients.

Output constancy was checked each month for all commissioned energies. The monthly output check for the 5.0 cm applicator was performed in a similar way to the daily output check but utilizing different QA equipment (slab phantoms: 30.0 cm × 30.0 cm × 4.0 cm [CNMC Virtual Water], 30.0 cm × 30.0 cm × 2.0 cm [PTW RW3 plate, water equivalent material with a density of 1.045 g/cm^3^]; ionization chamber: PTW TN23342A) (Figure 2b). The measured relative output for each energy was compared with the baseline established directly after absolute output calibration. The ionization chamber, PTW TN23342A, was chosen as this chamber was designed for low energy x‐rays. The applicator size and timer setting were selected for the same reason as for daily output constancy check. The monthly output constancy check ensures that outputs for all commissioned energies are consistent to deliver the intended radiation dose to patients.

#### 2.2.2 Backup timer (daily)

The backup timer is an interlock system which terminates an exposure when a problem with the main timer is indicated, and it counts the elapsed time of exposure. The backup timer was checked during daily output constancy check. After a selected beam was delivered, the backup timer value on the console was recorded and compared to the set timer value (0.3 min). This test ensures that there is no issue with the main timer and the main and backup timer indications agree.

#### 2.2.3 Timer accuracy (daily)

Timer accuracy was verified during daily output constancy check. The beam‐on duration was independently measured using a stopwatch and compared to the set timer value (0.3 min [18.0 sec]). This test ensures that the timer on the console computer is accurate to deliver the intended radiation dose to patients.

#### 2.2.4 Beam quality: first half value layer (HVL_1_) (annual)

**FIGURE 3:**
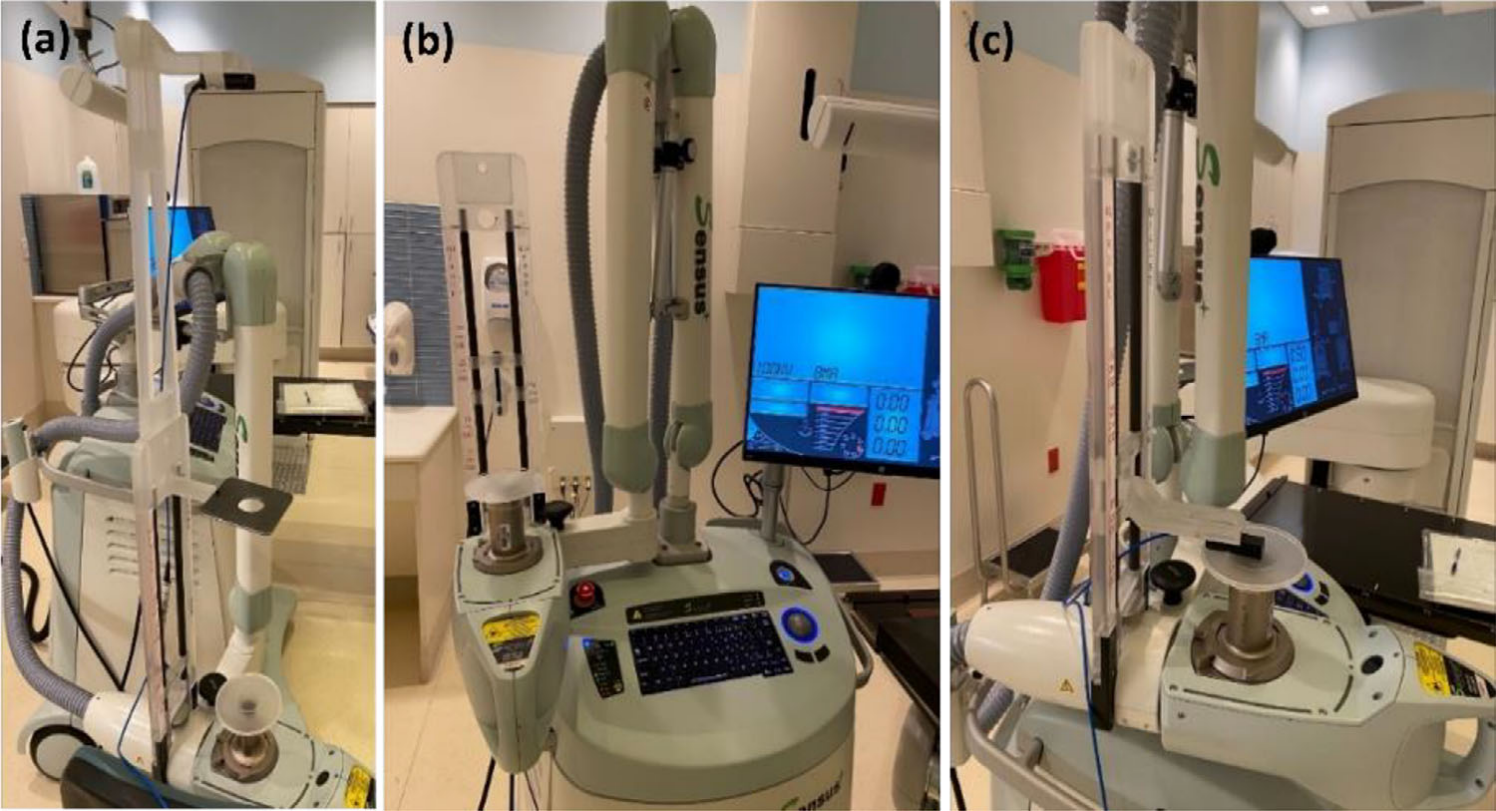
SRT‐100 setups for (a) half value layer (HVL), (b) applicator factor (AF), and (c) absolute output calibration.

HVL_1_ of an x‐ray beam is the thickness of a specified attenuator needed to reduce the air kerma rate in a narrow beam to half of its original value.^4^ HVL_1_ was measured annually for all commissioned energies. The 3.0 cm applicator, a lead platform with a 4.0 cm aperture and an ionization chamber (PTW TN23342A) were mounted at 15.0 cm, 50.0 cm, and 100.0 cm from the x‐ray source, respectively (Figure 3a). The polystyrene foil was placed on the applicator. An energy/filter combination (50 kV_p_/SRT50, 70 kV_p_/SRT70, or 100 kV_p_/SRT100) was selected and the timer was set to 0.3 min. Without an aluminum (Al) plate placed on top of the lead platform, the beam was delivered 3–4 times with the electrometer bias set to +300 V (MAX‐4000) and readings (rdgs) were recorded. Measurements were repeated with different thickness of Al plates. Al thickness to reach [rdgs]_Al_/[rdgs]_Al = 0 mm_ equal to 0.5 in a semi‐log plot was determined as HVL_1_ for each energy and compared with the baseline measured at commissioning. The ionization chamber and timer setting were selected for the same reason as for monthly output constancy check. The 3.0 cm applicator was selected to keep the same size used in the PTW calibration laboratory for HVL measurement. Annual verification of HVL ensures that beam quality of each energy does not change over time.

#### 2.2.5 Applicator factors (AFs) (annual)

AF is defined as output for each applicator (1.5, 2.0, 2.5, 3.0, or 4.0 cm applicator) relative to output for the reference applicator (5.0 cm applicator). AFs were measured annually for all commissioned applicators and energies. The 5.0 cm applicator and an ionization chamber (Standard Imaging Exradin A26) were mounted at 15.0 cm and 25.0 cm from the x‐ray source, respectively (Figure 3b). The polystyrene foil was placed on the applicator. Energy/filter (50 kV_p_/SRT50, 70 kV_p_/SRT70, or 100 kV_p_/SRT100) was selected and the timer was set to 0.3 min. The beam was delivered 3–4 times with the electrometer bias set to +300 V (MAX‐4000) and rdgs were recorded. Measurements were repeated for the rest of the applicators (1.5, 2.0, 2.5, 3.0, and 4.0 cm). AF was then determined as a ratio of [rdgs]_appl_ to [rdgs]_appl = 5.0 cm_ for each applicator and each energy and compared with the baseline measured at commissioning. The ionization chamber, Exradin A26, was used following the vendor's recommendation (Figure 3b). The timer setting was chosen for the same reason as output constancy check. AF measurements ensure that output for each applicator relative to output for the 5.0 cm applicator does not change over time to deliver the intended radiation dose to patients for a selected applicator.

#### 2.2.6 Absolute output calibration (annual)

Following the in‐air method of the American Association of Physicists in Medicine Task Group (AAPM TG)‐61 protocol^4^ absolute output calibration was verified annually for all commissioned energies. The 5.0 cm applicator, the polystyrene foil, and an ionization chamber (PTW TN23342A) were sequentially mounted at 15.0 cm from the x‐ray source (Figure 3c). Since the TN23342A chamber is vented to air, temperature and pressure of the room were recorded. Energy/filter (50 kV_p_/SRT50, 70 kV_p_/SRT70, or 100 kV_p_/SRT100) was selected and the timer was set to 0.5 min. The beam was delivered 3–4 times with the electrometer bias set to +300 V, +150 V and −300 V each (MAX‐4000), and rdgs (charges) were recorded for each bias voltage (raw rdgs [M_raw_] at +300 V). P_TP_ (temperature and pressure correction), P_ion_ (ionization chamber collection inefficiency correction), and P_pol_ (polarity correction) were calculated using the equations in the AAPM TG‐61 protocol.^4^ P_elec_, electrometer correction factor, was provided by an Accredited Dosimetry Calibration Laboratory (ADCL). For each energy, using the measured HVL_1_ value, the air‐kerma calibration factor ($N_{k}$) for the ionization chamber was interpolated from values provided by the ADCL. Similarly, backscatter factors ($B_{w}$), and the ratio for water‐to‐air of the mean mass energy absorption coefficients (${\left[{\left(\bar{\frac{\mu_{en}}{\rho }}\right)}_{air}^{w}\right]}_{air}$) were interpolated from values in Tables IV and V of AAPM TG 61.^4^ P_stem,air_, chamber stem correction factor, was assumed to be unity because of a minimal chamber stem difference between calibration and measurement. Absolute output (i.e., absorbed dose to water on the water surface [Gy/min]) for the 5.0 cm applicator was then determined using Equation (1) below^4^ for each energy and compared with the baseline measured during commissioning. Absolute outputs for the remaining applicators were determined using AFs and interpolated backscatter factors for each applicator, and were compared with the baselines at commissioning. The ionization chamber, PTW TN23342A, was used for the same reason as for monthly output constancy check. The 5.0 cm applicator was selected as the reference applicator because of the largest size commissioned. The timer was set to 0.5 min instead of 1.0 min to avoid rapid x‐ray tube heating due to longer exposure and a consequent interlock during measurements. Charges for 1.0 min for absolute output calibration were obtained from collected charges for 0.5 min multiplied by 2. The absolut output calibration check ensures that absolute output for each energy is within the tolerance and otherwise, it must be updated in the treatment unit and/or treatment time calculations.

(1) $$\begin{array}{cc} & D_{w,z=0}\left(Gy/min\right)=\left(M_{raw}\left[C/min\right]\cdot P_{elec}\cdot P_{TP}\cdot P_{ion}\cdot P_{pol}\right)\\ & \;\cdot N_{k}\left[\frac{Gy}{C}\right]\cdot B_{w}\cdot P_{stem,air}\cdot {\left[{\left(\bar{\frac{\mu_{en}}{\rho }}\right)}_{air}^{w}\right]}_{air} \end{array}$$

#### 2.2.7 Output constancy with varying x‐ray tube head rotation (annual)

**FIGURE 4:**
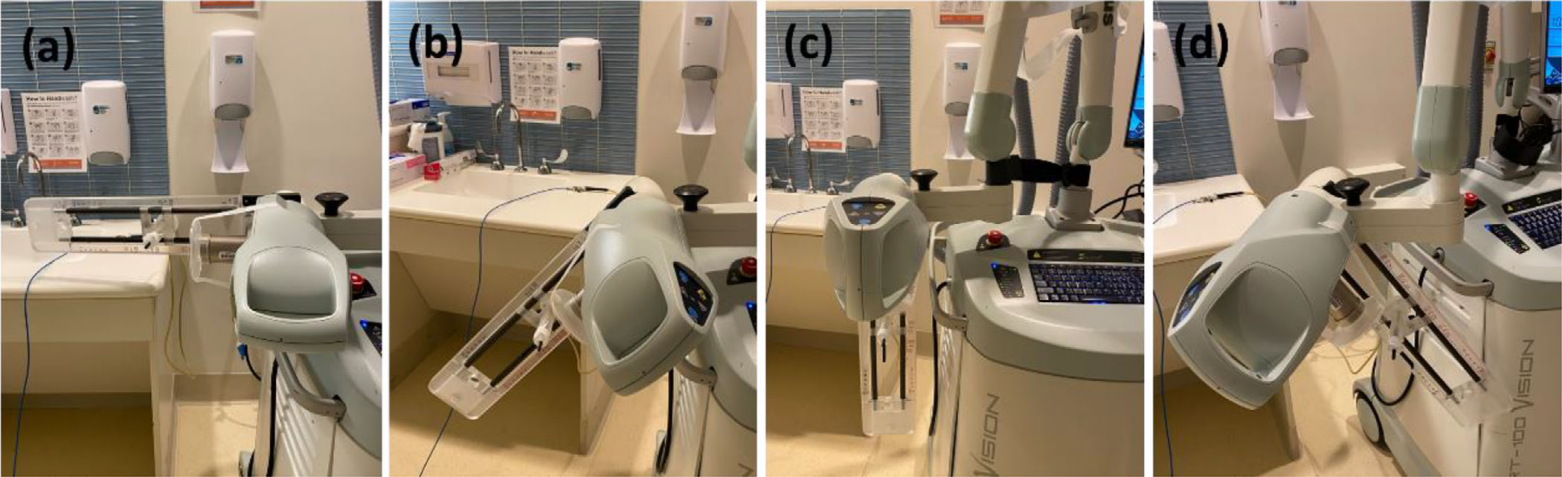
SRT‐100 setups for output constancy with the x‐ray tube head angled by (a) 90.0°, (b) 135.0°, (c) 180.0°, and (d) 225.0° counterclockwise from the head straight up (Figure 3b).

Output constancy with varying x‐ray tube head rotation was checked annually for all commissioned energies. Immediately following the AF measurements (x‐ray tube head of 0.0°, Figure 3b), using the same QA equipment and method, measurements for the 5.0 cm applicator were repeated with the tube head angled by 90.0°, 135.0°, 180.0° and 225.0° (Figure 4a–d). Outputs at 90.0°, 135.0°, 180.0°, and 225.0° of head angles were compared with the output at 0.0° for each energy. This test was implemented in 2024, and measurements were performed at four different time points over a 4‐week period. The head angles were selected based on clinical likelihood of use. This test ensures that outputs for various x‐ray tube head angles are consistent for different patient setups.

#### 2.2.8 Output reproducibility (annual)

Output reproducibility was assessed annually for all commissioned energies. With the same setup (5.0 cm applicator) and QA equipment as described for the AF measurements (Figure 3b), a beam for a timer setting of 0.3 min was delivered 10 times consecutively with the electrometer bias set to +300 V (MAX‐4000) and rdgs were recorded. Mean and standard deviation (SD) of 10 rdgs were calculated and output reproducibility was determined as SD/mean×100 (%)^1^ for each energy. This test verifies that output for the same timer setting is consistent each time a beam is delivered.

#### 2.2.9 Output linearity (annual)

Output linearity was verified annually for all commissioned energies. With the same setup (5.0 cm applicator) and QA equipment as for the AF measurements (Figure 3b), a beam for the timer of 0.1 min was delivered 3–4 times with the electrometer bias set to +300 V (MAX‐4000) and charges (pC) were collected. Measurements were repeated for timer settings of 0.2, 0.4, 0.6, 0.8, and 1.0 min. Charges (pC) were plotted as a function of timer (min), a linear regression line was generated, and a deviation (%) from the linear regression line for each timer point was determined for each energy. This test ensures that output is linear as a function of timer and no correction for nonlinearity is needed in treatment time calculations.

#### 2.2.10 Timer and end‐effect error (annual)

The timer and end‐effect error is defined as the amount of time not accounted for by the machine timer mechanism during the x‐ray beam delivery.^4^ It usually describes the time difference between when the machine timer starts and when the desired kV_p_ and mA have reached.^4^ One way of measuring the timer and end‐effect error is to use the graphical extrapolation method. Graphical extrapolation to zero exposure on an exposure versus timer plot yields the timer and end‐effect error.^4^

The timer and end‐effect error was measured annually for all commissioned energies. After output linearity was measured (Section 2.2.9), the linear regression line was extrapolated and the timer (min) for the charge of 0 pC was determined as the timer and end‐effect error for each energy. This test ensures that the timer and end‐effect error for the unit is determined and is accounted for in treatment time calculations if it is not negligible (> ± 0.01 min).

#### 2.2.11 Percent depth dose (PDD) verification: spot checks (annual)

Spot checks of PDD were performed annually for all commissioned applicators and energies. With the same setup (5.0 cm applicator) and QA equipment as for the monthly output constancy check (depth of 0.0 mm) shown in Figure 2b, a beam for the timer of 0.3 min was delivered 3–4 times with the electrometer bias set to +300 V (MAX‐4000) and rdgs were recorded. Measurements were repeated for depths of 1.0, 3.0, and 5.0 mm with slab phantom(s) (CNMC Virtual Water) added. Measurements were repeated for the other applicators. PDD was then determined as a ratio of [rdgs]_depth_ to [rdgs]_depth = 0 mm_ for each applicator and each energy, and was compared with the baseline measured during commissioning. The spot checks ensure that beam characteristics of each energy do not change over time.

#### 2.2.12 Congruence between radiation field and applicator size at FSD (annual)

Congruence between radiation field and applicator size at FSD was verified on an annual basis for all commissioned applicators. A slab phantom (size: 30.0 cm × 30.0 cm × 4.0 cm, CNMC Virtual Water), a Gafchromic film (EBT3, Ashland LLC, Bridgewater, NJ) and the polystyrene foil were placed on a table in sequence (Figure 2c). The 5.0 cm applicator attached to the tube head was centered on the film. After energy/filter (100 kV_p_/SRT100) was selected and the timer was set to 0.3 min, the beam was delivered. Measurements were repeated for all remaining applicators. After 24 h, films were scanned with 300 dots per inch using an Epson 10000XL scanner (Seiko Epson Corp., Nagano, Japan). In RIT software (Radiological Imaging Technology, Inc., Colorado Springs, CO), full widths at half maximum (FWHMs) were measured in both the cathode–anode direction and its perpendicular direction. The average value of the FWHMs in the two directions was compared to the nominal applicator size for each applicator. 100 kV_p_/SRT100 was selected after a minimal difference (≤ ± 0.2 mm) in congruence among three energies for all six applicators was confirmed at the time of commissioning. This test ensures that a selected applicator size matches the intended radiation field size at FSD.

### 2.3 Mechanical tests

#### 2.3.1 Mechanical stability and safety (monthly)

Mechanical stability and safety were checked each month. It was verified that the unit and associated accessories were firmly anchored and could be used without harming patients or staff. This test ensures safety of the unit for patients and staff.

#### 2.3.2 Applicator integrity and indicators (monthly)

Applicator integrity and indicators were checked every month. Each applicator was visually inspected for any damage or change. Also, each applicator was mounted and verified with the indicator shown in the base unit display. This test ensures that all applicators are physically sound, and the unit recognizes a mounted applicator correctly to deliver the intended radiation dose to patients.

### 2.4 Safety tests

#### 2.4.1 Safety tests (daily)

Safety features were tested on the day of treatment. The functionality of both cameras in the unit and the monitor on the console was checked. The audio system in the unit was tested. The beam‐on indicator outside the room was verified during beam delivery. Additionally, interlock systems including the door, key, and beam‐off button were tested in beam‐on conditions. These tests ensure that safety features of the unit are functional for treatment.

#### 2.4.2 Energy/filter indicators (daily)

Energy/filter indicators were checked daily for all commissioned energy. Using a QA plan consisting of three fields with each energy (50 kV_p_, 70 kV_p_, or 100 kV_p_), each field was loaded individually, and the corresponding energy/filter indicator was confirmed on the control console display. This test ensures that a correct combination of energy and filter is selected in the treatment unit to use the intended energy with correct beam quality for patients.

### 2.5 Imaging tests

#### 2.5.1 Functional check (daily)

The functional check of the ultrasound was performed on the day of use. Unlike other commercial ultrasound probes, the probe of the SRT‐100 Vision unit has an empty space inside which must contain an acoustic absorber (i.e., transmission medium) before use. Each day the empty space of the probe was filled with distilled water first. The transducer of the probe was then activated, and its operation was tested under the ultrasound module.

#### 2.5.2 Spatial integrity: measurement accuracy (monthly)

**FIGURE 5:**
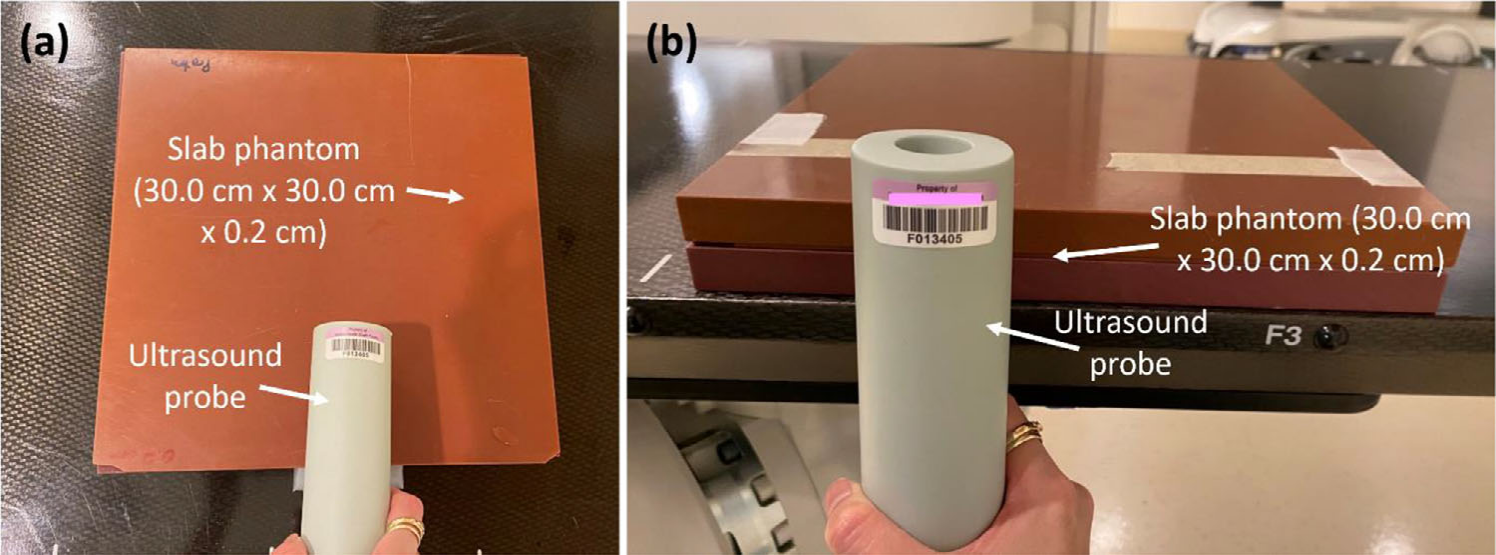
Setups for (a) depth and (b) width measurements on ultrasound images acquired in an SRT‐100 Vision unit.

Two‐dimensional (2D) measurement accuracy was verified on ultrasound images monthly. The ultrasound probe was filled with distilled water. A slab phantom (size: 30.0 cm × 30.0 cm × 0.2 cm, CNMC Virtual Water) was placed on a table and ultrasound gel was applied on the phantom surface (Figure 5a). Under the ultrasound module of the unit, an ultrasound image of the phantom was acquired by scanning the phantom from the surface (Figure 5a). The depth of the phantom was measured using the digital measurement tool. After a speed of sound correction (= [1540 m/sec, soft tissue for which ultrasound is calibrated]/[1560 m/sec, slab phantom with a density of 1.04 g/cm^3^])^12^ was applied to the measured depth, the result was compared with the known value (2.0 mm). Then the same slab phantom was sandwiched between two thicker slab phantoms (size: 30.0 cm × 30.0 cm × 2.0 cm, CNMC Virtual Water) on the table such that the 2.0‐mm thick phantom was indented between the other two phantoms to separate the 2.0‐mm phantom from the other phantoms (Figure 5b). Ultrasound gel was applied on the side of the phantom and an image of the phantom was acquired by scanning the phantom from the side (Figure 5b). The thickness (width) of the 2.0‐mm phantom was measured, a speed of sound correction was applied, and the result was compared with the known value (2.0 mm). This test ensures that the ultrasound was properly calibrated to measure the size of a lesion accurately on an ultrasound image.

#### 2.5.3 Image quality: artifact (monthly)

Image quality was verified monthly. Ultrasound images of the slab phantom were reviewed and any artifact on the images was visually checked. This test ensures that image quality is adequate to see a lesion on an ultrasound image.

## 3 RESULTS

The three‐ and half‐year results of the periodic machine QA tests for the SRT‐100 Vision unit are presented below:

### 3.1 Warm‐up

#### 3.1.1 Machine warm‐up (daily)

Machine warm‐up was successfully completed, and output recalibration was not required on any of the treatment days.

### 3.2 Dosimetry tests

#### 3.2.1 Output constancy (daily, monthly)

**FIGURE 6:**
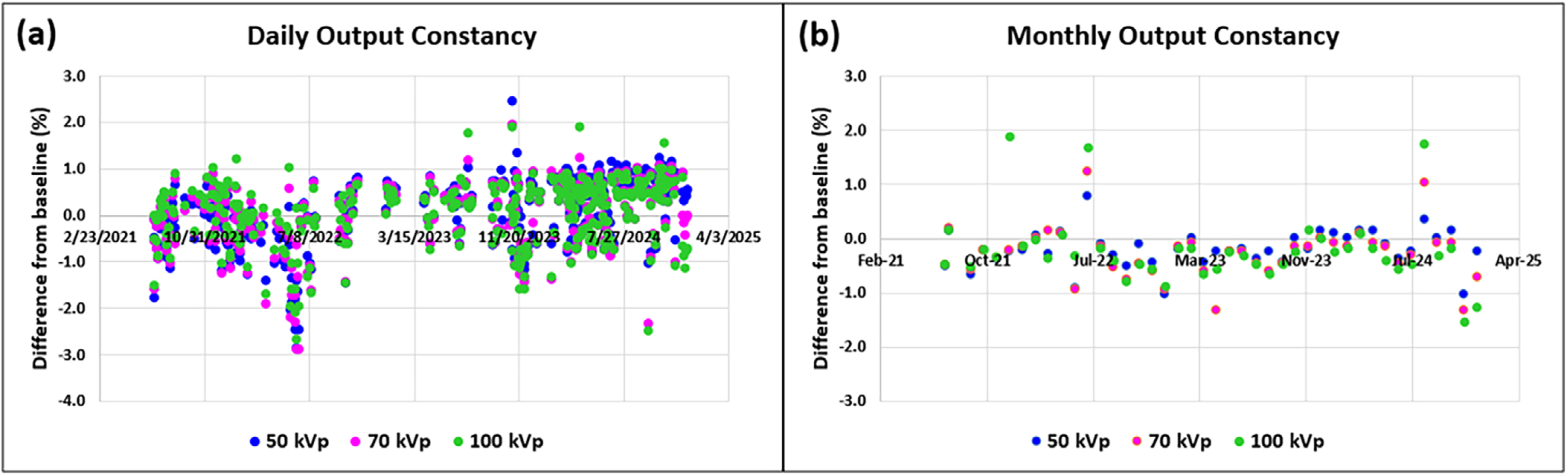
First three‐ and ‐half year results of (a) daily output constancy and (b) monthly output constancy for three energies of an SRT‐100 Vision unit.

Daily outputs were < ± 3.0% from the baselines and were within the tolerance (baseline ± 3.0%) for all three energies (Figure 6a). Mean ± SD (min, max) daily output differences (%) from the baselines were 0.1% ± 0.7% (−2.9%, 2.4%), 0.1% ± 0.7% (−2.9%, 2.0%) and 0.1% ± 0.7% (−2.7%, 1.9%) for 50, 70, and 100 kV_p_, respectively.

Monthly outputs were < ± 2.0% from the baselines and were within the tolerance (baseline ± 3.0%) for all three energies (Figure 6b). Mean ± SD (min, max) monthly output differences (%) from the baselines were ‐0.2% ± 0.3% (−1.0%, 0.8%), ‐0.3% ± 0.5% (−1.3%, 1.2%) and ‐0.2% ± 0.7% (−1.5%, 1.9%) for 50, 70, and 100 kV_p_, respectively.

#### 3.2.2 Backup timer (daily)

On each day of treatment, the backup timer consistently displayed 0.3 min matching the preset timer value of 0.3 min (mean difference of 0.0% from the timer setting), meeting the required tolerance (timer set ± 5.0%).

#### 3.2.3 Timer accuracy (daily)

Mean ± SD (min, max) beam‐on duration measured for the timer of 0.3 min (18.0 sec) was 17.8 s ± 0.3 s (17.4 s, 18.8 s) and was within the tolerance (timer set ± 1.0% or ± 1.0 s, whichever is greater).

#### 3.2.4 Beam quality: HVL_1_ (annual)

**FIGURE 7:**
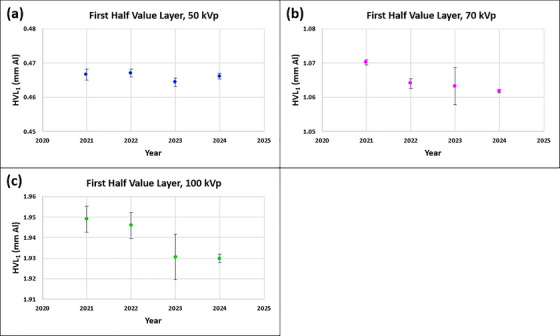
First half value layers (HVLs) measured over three years for (a) 50 kV_p_, (b) 70 kV_p_, and (c) 100 kV_p_ of an SRT‐100 Vision unit.

Mean ± SD (min, max) HVLs in mm Al measured over three years were 0.47 mm ± 0.00 mm (0.46 mm, 0.47 mm), 1.06 mm ± 0.00 mm (1.06 mm, 1.06 mm), and 1.94 mm ± 0.01 mm (1.93 mm, 1.95 mm) for 50, 70, and 100 kV_p_, respectively (Figure 7). Corresponding differences (%) from the baselines (year of 2021) were −0.3% ± 0.3% (−0.6%, 0.0%), −0.7% ± 0.1% (−0.7%, −0.6%) and ‐0.7% ± 0.5 (−1.0%, −0.2%) and were well below the tolerance (baseline ± 10.0%).

#### 3.2.5 AFs (annual)

**FIGURE 8:**
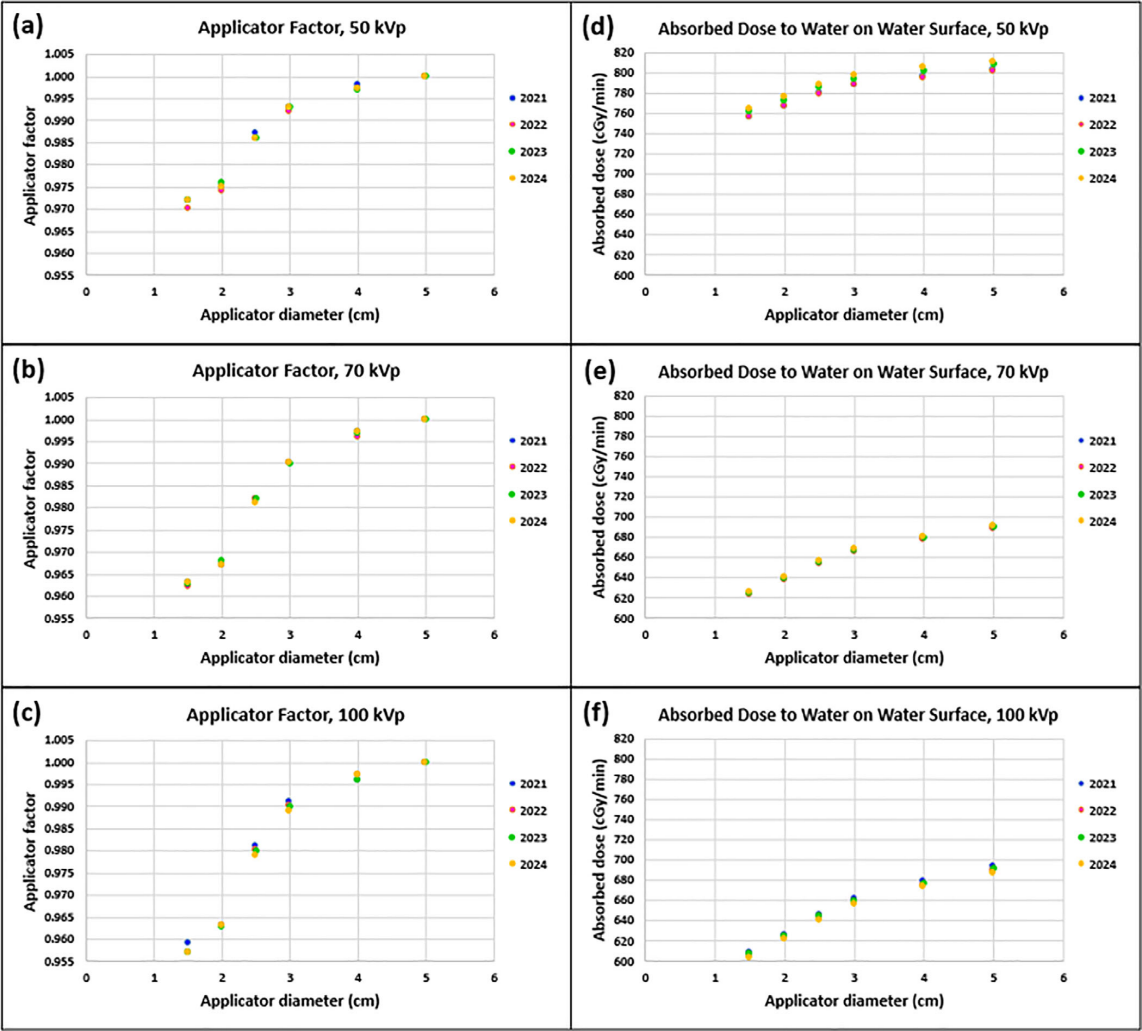
(a)–(c) Applicator factors (AFs) and (d)–(f) absolute outputs (absorbed dose to water on the water surface [cGy/min]) for six applicators measured for three energies of an SRT‐100 Vision unit over a three‐year period. Error bars are omitted due to the coarse resolution in the vertical axis. Tables A and B in supplementary material present mean and standard deviations (SDs) instead.

AFs measured for a 3‐year period are plotted in Figure 8a–c. The measurements were all within the tolerance (baseline ± 3.0%) for all three energies. Differences (%) of measured AFs from the baselines (year of 2021) for three energies are tabulated in Table 3.

**TABLE 3:** Differences (%) of applicator factors and absolute outputs measured over three years from the baselines for three energies.

		Differences (%) of applicator factors from the baselines					
		1.5 cm	2.0 cm	2.5 cm	3.0 cm	4.0 cm	5.0 cm
**50 kV_p_**	**Mean **± **SD (%)**	0.0 ± 0.2	0.0 ± 0.2	0.0 ± 0.1	0.0 ± 0.1	0.0 ± 0.1	0.0 ± 0.0
	**Min (%)**	−0.2	−0.1	−0.1	−0.1	−0.1	0.0
	**Max (%)**	0.2	0.2	0.0	0.1	0.0	0.0
**70 kV_p_**	**Mean **± **SD (%)**	0.0 ± 0.1	0.0 ± 0.1	0.0 ± 0.1	0.0 ± 0.0	0.0 ± 0.1	0.0 ± 0.0
	**Min (%)**	−0.1	−0.1	−0.1	0.0	−0.1	0.0
	**Max (%)**	0.1	0.1	0.0	0.0	0.1	0.0
**100 kV_p_**	**Mean **± **SD (%)**	−0.1 ± 0.1	0.0 ± 0.0	−0.1 ± 0.1	−0.1 ± 0.1	0.0 ± 0.1	0.0 ± 0.0
	**Min (%)**	−0.2	0.0	−0.1	−0.1	−0.1	0.0
	**Max (%)**	0.0	0.0	0.0	0.0	0.1	0.0

		Differences (%) of absolute outputs from the baselines					
		1.5 cm	2.0 cm	2.5 cm	3.0 cm	4.0 cm	5.0 cm
**50 kV_p_**	**Mean **± **SD (%)**	0.6 ± 0.6	0.6 ± 0.6	0.6 ± 0.6	0.6 ± 0.6	0.6 ± 0.6	0.6 ± 0.6
	**Min (%)**	−0.1	−0.1	−0.1	−0.1	−0.1	−0.1
	**Max (%)**	1.2	1.1	1.2	1.1	1.1	1.1
**70 kV_p_**	**Mean **± **SD (%)**	0.0 ± 0.2	0.0 ± 0.2	0.0 ± 0.2	0.0 ± 0.3	0.0 ± 0.3	0.0 ± 0.2
	**Min (%)**	−0.3	−0.2	−0.2	−0.3	−0.3	−0.3
	**Max (%)**	0.2	0.2	0.2	0.2	0.2	0.1
**100 kV_p_**	**Mean **± **SD (%)**	−0.5 ± 0.3	−0.5 ± 0.3	−0.5 ± 0.3	−0.5 ± 0.3	−0.5 ± 0.3	−0.5 ± 0.3
	**Min (%)**	−0.8	−0.8	−0.9	−0.8	−0.8	−0.8
	**Max (%)**	−0.2	−0.2	−0.2	−0.3	−0.2	−0.2

Abbreviations: kVp: peak kilovoltage; SD, standard deviation.

#### 3.2.6 Absolute output calibration (annual)

Absolute outputs measured from 2021 to 2024 are shown in Figure 8d–f. Measured absolute outputs were consistent with the baselines (year of 2021) within ± 1.2% (tolerance: ± 3.5% from baseline) for all three energies. Table 3 presents differences (%) of measured absolute outputs from the baselines for three energies.

#### 3.2.7 Output constancy with varying x‐ray tube head rotation (annual)

**FIGURE 9:**
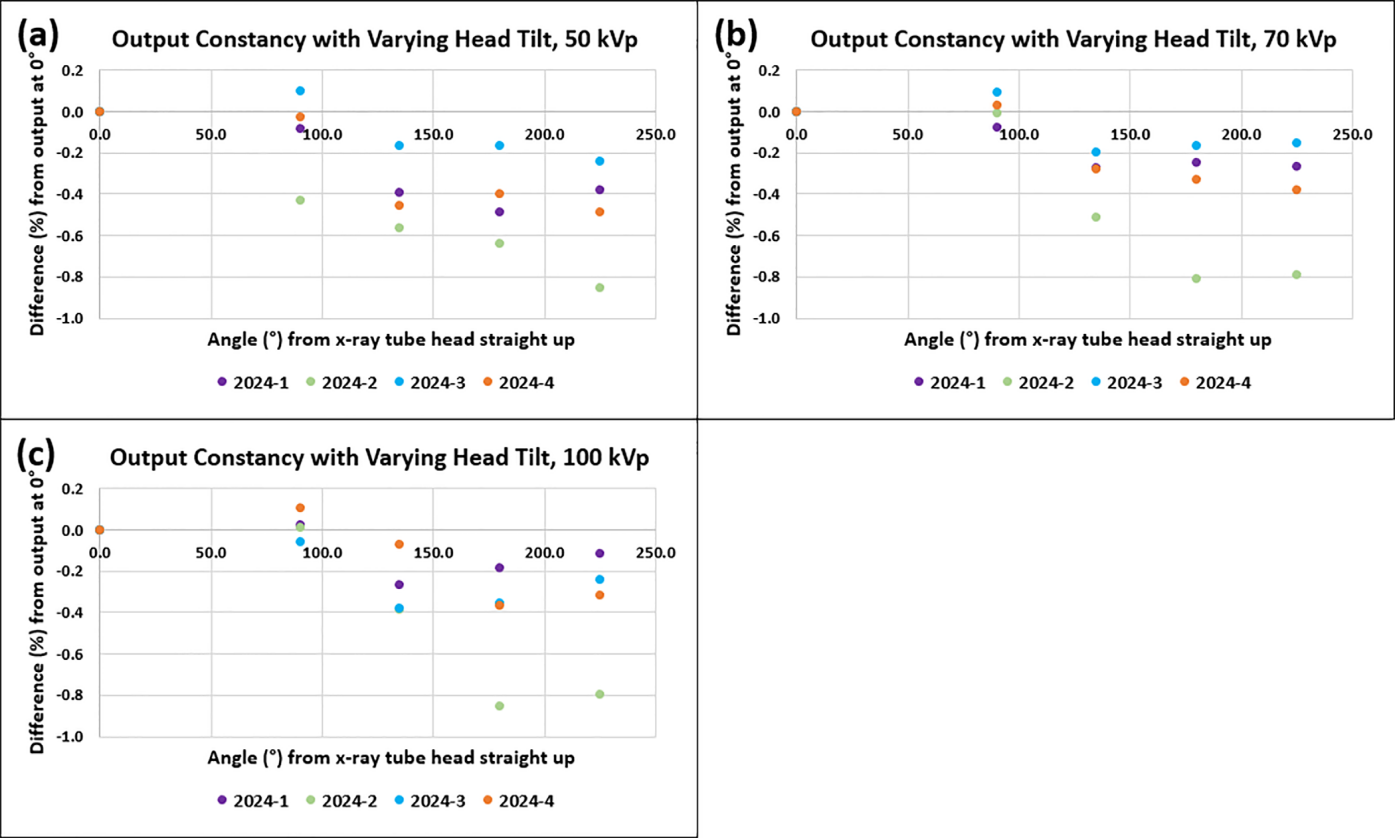
Output constancy with varying x‐ray tube head rotation measured for three energies of an SRT‐100 Vision unit at four different time points in 2024.

Figure 9 displays differences (%) of the outputs for varying head rotation from the outputs for head straight up measured at four different time points in 2024. For 50 kV_p_, mean ± SD (min, max) output differences (%) were −0.1% ± 0.2% (−0.4%, 0.1%), −0.4% ± 0.2% (−0.6%, −0.2%), −0.4% ± 0.2% (−0.6%, −0.2%), and −0.5% ± 0.3% (−0.9%, −0.2%) for 90.0°, 135.0°, 180.0°, and 225.0° of head rotations, respectively. Corresponding differences (%) were 0.0% ± 0.1% (−0.1%, 0.1%), −0.3% ± 0.1% (−0.5%, −0.2%), −0.4% ± 0.3% (−0.8%, −0.2%), and −0.4% ± 0.3% (−0.8%, −0.2%) for 70 kV_p_ and were 0.0% ± 0.1% (−0.1%, 0.1%), −0.3% ± 0.1% (−0.4%, −0.1%), −0.4% ± 0.3% (−0.9%, −0.2%), and −0.4% ± 0.3% (−0.8%, −0.1%) for 100 kV_p_. These measurements were below the set tolerance (± 2.0% from output with head straight).

#### 3.2.8 Output reproducibility (annual)

Mean ± SD (min, max) output reproducibility was 0.1% ± 0.0% (0.0%, 0.1%), 0.1% ± 0.0% (0.0%, 0.1%) and 0.0% ± 0.0% (0.0%, 0.1%) for 50, 70, and 100 kV_p_, respectively, which were well below the tolerance (2.0%).

#### 3.2.9 Output linearity (annual)

**FIGURE 10:**
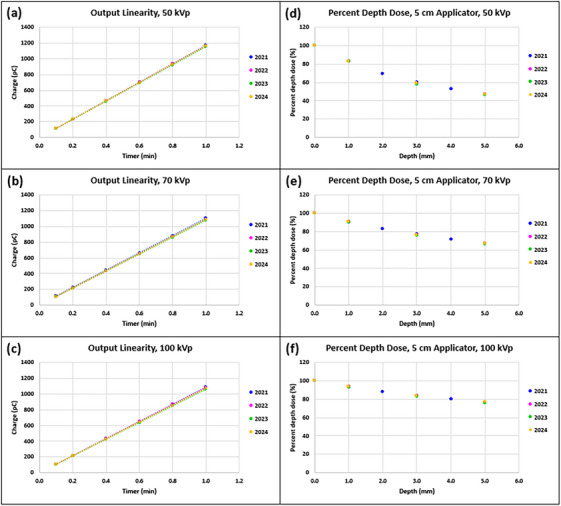
(a)–(c) Output linearity and (d)–(f) percent depth dose (PDD) for the 5.0 cm applicator measured for three energies of an SRT‐100 Vision unit over the course of three years. Error bars are omitted due to the coarse resolution in the vertical axis. Tables C and D in supplementary material present mean and standard deviations (SDs) instead.

Figure 10a–c shows output linearity measured over 3 years. For all three energies, the maximum deviations (%) from the linear regression lines occurred at the timer of 0.1 min but were within the tolerance (± 1.0%). Deviations (%) of measurements from the linear regression lines at timer settings of 0.1, 0.2, 0.4, 0.6, 0.8, and 1.0 min for three energies are presented in Table 4.

**TABLE 4:** Deviations (%) of measurements over three years from linear regression lines for three energies.

		0.1 min	0.2 min	0.4 min	0.6 min	0.8 min	1.0 min
**50 kV_p_**	**Mean **± **SD (%)**	−0.2 ± 0.3	−0.2 ± 0.2	−0.2 ± 0.2	−0.1 ± 0.2	−0.1 ± 0.2	−0.1 ± 0.2
	**Min (%)**	−0.5	−0.3	−0.3	−0.3	−0.3	−0.4
	**Max (%)**	0.1	0.0	0.0	0.0	0.0	0.0
**70 kV_p_**	**Mean **± **SD (%)**	0.1 ± 0.1	0.0 ± 0.0	0.0 ± 0.0	0.0 ± 0.0	0.0 ± 0.0	0.0 ± 0.0
	**Min (%)**	0.0	0.0	0.0	−0.1	0.0	0.0
	**Max (%)**	0.1	0.0	0.0	0.0	0.0	0.0
**100 kV_p_**	**Mean **± **SD (%)**	−0.1 ± 0.2	0.0 ± 0.0	0.0 ± 0.1	0.0 ± 0.1	0.0 ± 0.0	0.0 ± 0.0
	**Min (%)**	−0.2	−0.1	−0.1	0.0	0.0	0.0
	**Max (%)**	0.2	0.0	0.1	0.1	0.0	0.0

Abbreviations: kVp: peak kilovoltage; SD, standard deviation.

#### 3.2.10 Timer and end‐effect error (annual)

Mean ± SD (min, max) timer and end‐effect errors extrapolated from the linear regression lines were ‐0.0002 min ± 0.0001 min (−0.0003 min, −0.0001 min), 0.0000 min ± 0.0001 min (−0.0001 min, 0.0001 min), and −0.0002 min ± 0.0003 min (−0.0005 min, 0.0001 min) for 50, 70, and 100 kV_p_, respectively. These results were well below the tolerance (± 0.01 min).

#### 3.2.11 PDD verification: spot checks (annual)

The results of PDD spot checks at depths of 1.0, 3.0, and 5.0 mm for the 5.0 cm applicator showed that the differences from the baselines (year of 2021) were less than ± 2.2% (Figure 10d–f) for all three energies. For 50 kV_p_, mean ± SD (min, max) absolute differences from the baselines were 0.1% ± 0.1% (0.0%, 0.2%), −1.8% ± 0.3% (−2.1%, −1.5%) and −0.2% ± 0.3% (−0.5%, 0.0%) for depths of 1.0, 3.0 and 5.0 mm, respectively. Corresponding differences were 0.2% ± 0.0% (0.1%, 0.2%), −1.2% ± 0.4% (−1.6%, ‐0.9%) and −0.2% ± 0.3% (−0.6%, 0.0%) for 70 kV_p_ and were 0.1% ± 0.1% (0.0%, 0.3%), ‐1.0% ± 0.4% (−1.4%, ‐0.6%) and −0.4% ± 0.4% (−0.8%, −0.1%) for 100 kV_p_. PDDs for the rest of the applicators measured over three years were also within the tolerance (baseline ± 3.0%) (data not shown here).

#### 3.2.12 Congruence between radiation field and applicator size at FSD (annual)

**FIGURE 11:**
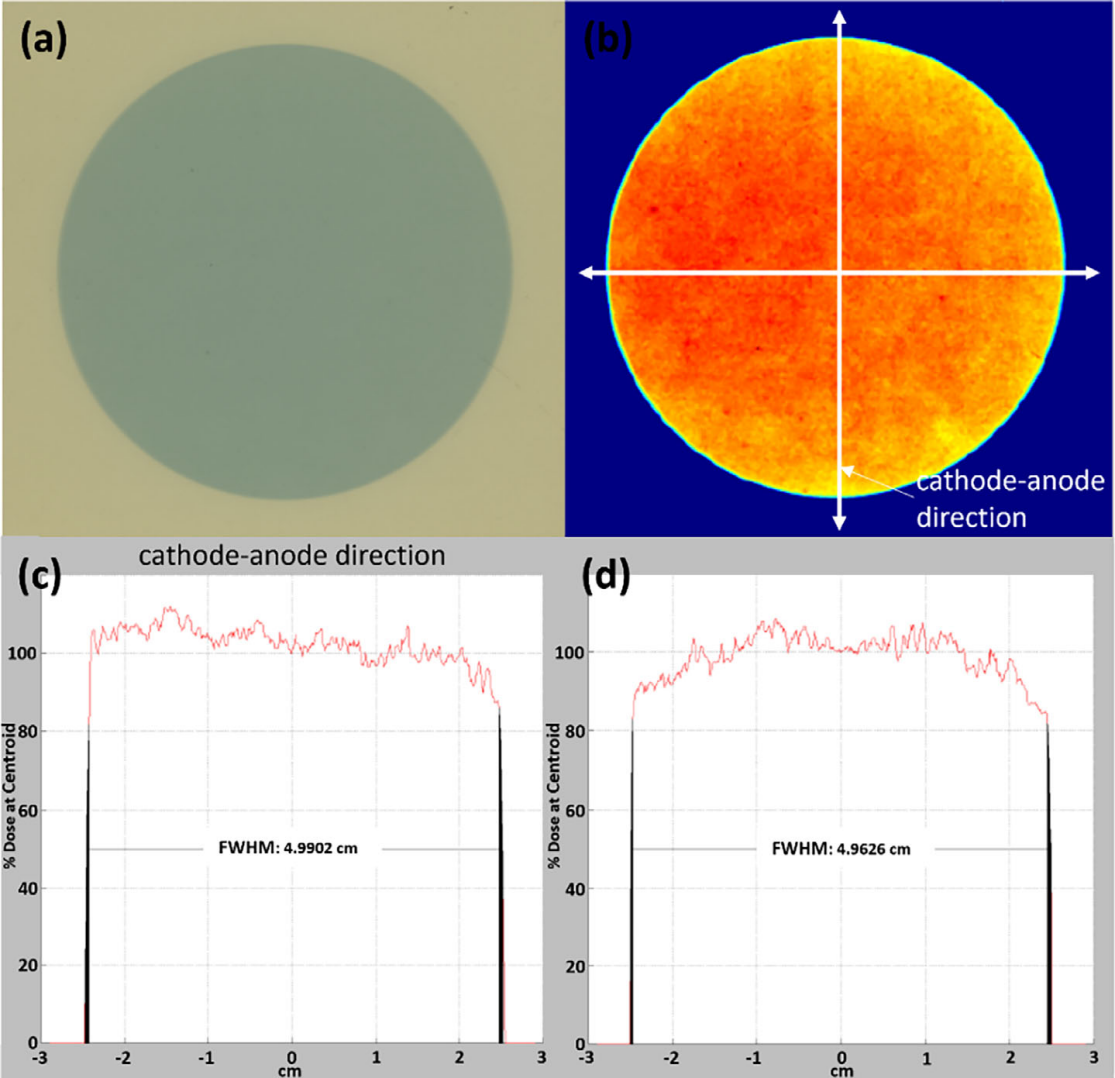
(a) Irradiated film, (b) scanned film with dose calibration applied,(c) profile in the cathode‐anode direction, and (d) profile in perpendicular to the cathode‐anode direction.

Congruence for all commissioned applicators was well below the tolerance (± 2.0 mm). Mean ± SD (min, max) absolute differences between measured FWHMs of radiation fields and nominal applicator diameters were 0.0 mm ± 0.2 mm (−0.1 mm, 0.3 mm), −0.2 mm ± 0.1 mm (−0.4 mm, −0.1 mm), −0.2 mm ± 0.2 mm (−0.4 mm, 0.0 mm), −0.1 mm ± 0.0 mm (−0.1 mm, 0.0 mm), −0.2 mm ± 0.1 mm (−0.2 mm, −0.1 mm) and −0.3 mm ± 0.1 mm (−0.4 mm, ‐0.2 mm) for 1.5, 2.0, 2.5, 3.0, 4.0, and 5.0 cm, respectively. Figure 11 shows an example of an irradiated film and film analysis results for the 5.0 cm applicator.

### 3.3 Mechanical tests

#### 3.3.1 Mechanical stability and safety (monthly)

Each month it was confirmed that the unit, treatment arms, and x‐ray tube were securely and safely anchored. No safety incidents were reported during this period.

#### 3.3.2 Applicator integrity and indicators (monthly)

Monthly inspections confirmed no damage or physical change to any applicator. Additionally, applicator indicators were verified to be functional for all applicators.

### 3.4 Safety tests

#### 3.4.1 Safety tests (daily)

All safety features and interlock systems were verified to be functional on each treatment day.

#### 3.4.2 Energy/filter indicators (daily)

Energy/filter indicators were verified to be functional.

### 3.5 Imaging tests

#### 3.5.1 Functional check (daily)

The ultrasound probe and ultrasound module were functional on each day of use during the evaluation period.

#### 3.5.2 Spatial integrity: measurement accuracy (monthly)

**FIGURE 12:**
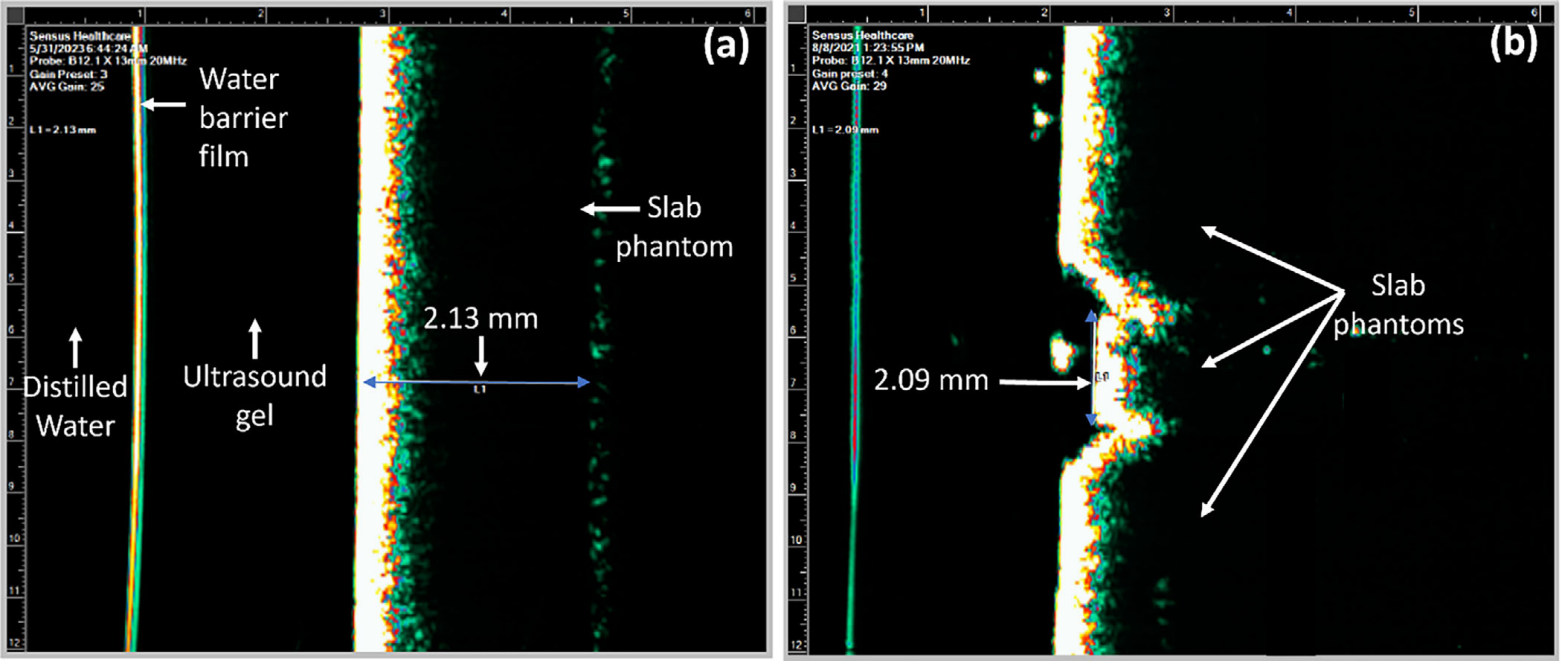
(a) Depth and (b) width measurements of a slab phantom on ultrasound images acquired in an SRT‐100 Vision unit.

Depth and width measured on ultrasound images for three and a half years were all within ± 0.4 mm from the reference values (2.0 mm). Mean ± SD (min, max) depth and width were 2.0 mm ± 0.1 mm (1.9 mm, 2.3 mm) and 2.1 mm ± 0.1 mm (2.0 mm, 2.4 mm), respectively and mean (min, max) absolute differences from the reference values were 0.0 mm (−0.1 mm, 0.3 mm) and 0.1 mm (0.0 mm, 0.4 mm), achieving the tolerance (± 1.0 mm) each month. Figure 12 shows an example of depth and width measurements on ultrasound images.

#### 3.5.3 Image quality: artifact (monthly)

No significant artifact was observed on ultrasound images acquired each month (Figure 12).

## 4 DISCUSSION

The results of relative and absolute output measurements demonstrated that machine outputs of our SRT‐100 Vision unit for the first three‐ and half‐year were stable within tolerances for the three energies commissioned (Figures 6 and 8d–f). For QA efficiency, we implemented daily output check only for the energy used on the day of treatment. In contrast, monthly output check covers all three commissioned energies. Absolute output results can vary from detector to detector^6^ and therefore, the same setup and the same detector (vendor and model) used during commissioning must be utilized and detector‐specific outputs must be compared annually. When detector(s) have new calibration factor(s) from an ADCL or are back from repairs, absolute outputs must be verified using the new factor(s), and baselines for relative outputs must be checked and be re‐established if needed. Since the absolute outputs measured at commissioning (baselines) have been consistent within ± 1.2%, those entered at the unit and in our time calculation worksheet were kept unchanged. This constancy was also confirmed with daily output calibration checks by RADCheck (diode) (< 3.0%) for three and a half years.

Two different ionization chambers were used for output constancy checks. For daily output constancy check, a thin entrance window ionization chamber (PTW TN34045) designed for electron beams was used in a slab phantom to avoid the use of the same ionization chamber (low energy x‐ray chamber) for monthly output constancy check. Hill et al. investigated the TN34045 chamber for relative dosimetry of kV x‐ray energies ranging from 50 kV_p_ (HVL of 1.6 mm Al) to 280 kV_p_ (HVL of 3.3 mm Cu).^13^ Although they concluded that the TN34045 chamber was suitable for direct depth dose measurements of kV x‐ray energies without correction factors within an accuracy of 3% difference compared to Monte Carlo (MC) data, a deviation of surface dose (depth of 0.0 mm) measured using the chamber from MC data was greater than 5.0% for 50 kV_p_.^13^ Therefore, our daily relative outputs measured at a depth of 0.0 mm using the TN34045 chamber have an unknown uncertainty and could have been underestimated.^13^ Knowing this, in‐air measurement using another detector suitable for our kV x‐ray energy ranges will be implemented in our daily QA program. Since the TN34045 chamber is vented to air, the baselines were established over multiple days from an average value of relative outputs with a temperature and pressure correction after absolute output calibration was performed. On each day of treatment, temperature and pressure corrected daily outputs relative to baselines were checked. For monthly output constancy and absolute output calibration checks, an ultrathin entrance window ionization chamber (PTW TN23342A) was used because it was primarily constructed for relative and absolute dosimetry of low kV x‐rays. This chamber is vented to air as well and therefore, requires a temperature and pressure correction.

For QA efficiency, timer accuracy check was done in conjunction with daily output check for the timer setting of 0.3 min (18.0 sec). However, 18.0 s seemed short, and the tolerance of timer set ± 1.0 sec needed to be applied. For longer timer settings, for instance, 1.0 min (60.0 s), the tolerance of timer set ± 1.0% could be achieved.

A detector response can be energy dependent in kV x‐ray energies,^13^ and a detector with sufficient energy independence must be used for HVL measurement.^4^ Since the TN23342A chamber has relatively a flat energy response up to 100 kV_p_,^14^ the energy response was not accounted for in HVL measurements (i.e., HVLs were measured from electrometer rdgs). According to the TG‐61 protocol, however, HVL should be measured from air kerma rate (ratio).^4^ Air kerma rate, K_air_, is defined in Equation (2) below. In M_corr_ (corrected electrometer rdgs), P_ele_∙P_TP_∙P_ion_∙P_pol_ would be constant if temperature and pressure of the room stay the same during measurements. According to the calibration certificate, the air kerma calibration factor, N_k_, for our TN23342 chamber varies by 0.74% for energy < 70 kV_p_ and is constant in the energy range of 70–100 kV_p_. Therefore, there is no inconsistency in HVL between charge/current based measurements and air kerma rate based measurements and no difference in reported absolute outputs for 70 kV_p_ and 100 kV_p_. For 50 kV_p_, HVL_1_ (first HVL) is 0.47 mm and HVL_2_ (second HVL) is 1.35 mm. Considering a slight beam quality change with filtration based on the air kerma calibration factor, corrected HVL_1_ is smaller only by 0.005 mm. This small change to HVL_1_ would not affect backscatter factors, the ratio of the mean mass energy absorption coefficients and absolute outputs. In the current work, HVL_1_ was measured and reported as a beam quality indicator. Future annual QA cycles will also include measurement of HVL_2_ to evaluate a degree of a beam quality change with filtration.

(2) $$\begin{array}{cc} & K_{\mathrm{air}}\left(\mathrm{Gy}/\sec\right)=M_{\mathrm{corr}}\left(C/\sec\right)\cdot N_{k}\left(\mathrm{Gy}/C\right)\\ & \;=\left(M_{\mathrm{raw}}\cdot P_{\mathrm{ele}}\cdot P_{\mathrm{TP}}\cdot P_{\mathrm{ion}}\cdot P_{\mathrm{pol}}\right)\cdot N_{k} \end{array}$$

where K_air_ (Gy/sec) is air kerma rate, M_corr_ (C/sec) is corrected electrometer rdg (current), M_raw_ (C/sec) is uncorrected electrometer rdg (current), P_ele_ is the electrometer correction factor, P_ion_ is the ion recombination correction factor, P_pol_ is the polarity correction factor, and N_k_ (Gy/C) is the air kerma calibration factor for a specified x‐ray beam quality.

HVL measurements are essential not only to check beam quality but also to interpolate air kerma calibration factors, backscatter factors, and mean mass energy absorption coefficients used for absolute output calibration of the AAPM TG‐61 protocol. According to AAPM TG 61, depending on the chamber's energy dependence, significant errors may occur in interpolation between lightly filtered (L series) and medium filtered (M series) beams and interpolation may be performed within the same series (L or M) of x‐ray beams only.^4^ The energies (50–100 kV_p_) that we commissioned belong to M series and interpolation of air kerma calibration factors was performed after HVL measurement.

AFs measured over 3 years did not change substantially (< ± 0.3% from the baselines at commissioning) confirming the robust design of the applicators. Therefore, routine measurements of AFs may be omitted from annual QA unless physical damage or structural changes to the applicators are suspected, in which case AFs must be measured to verify integrity and no change in AF values.

Output constancy with varying x‐ray tube head rotation was recently implemented following the most recent Canadian guidelines.^10^ The x‐ray tube head is typically rotated during treatments, and outputs at clinically relevant angles must be accessed. Measurement results over four weeks demonstrated consistent outputs (< ± 1.0%) in clinically used head angle ranges.

Output as a function of timer was linear (< ± 0.6%) and timer and end‐effect errors were negligible (< ± 0.0006 min) for all three energies. If the timer and end‐effect error is not negligible (i.e., > ± 0.01 min [timer resolution of our SRT‐100 Vision unit]), it can cause a systematic error in dose delivery and therefore, it must be accounted for in treatment time calculations (Equation [1]).

PDD measurements for low kV energies can be challenging. The use of a water phantom is not feasible for Sensus Healthcare units due to the x‐ray tube design (Figure 1a) and a reproducible setup with solid water phantoms is susceptible to inconsistent measurements. When solid water phantoms are used for PDD measurements, a correct selection is crucial because not all solid water phantoms are considered water equivalent and are recommended for relative dosimetry of low energy x‐ray beams.^15^ According to the manufacturer, the slab phantoms (CNMC Virtual Water) that we used for PDD measurements scatter and attenuate diagnostic and radiotherapy range x‐rays in the same way as water.^11^ Over 3 years, spot checks of PDDs were performed for all six applicators and the results were consistent within ± 2.1% of baseline values. Therefore, limiting spot checks of PDD for the 5.0 cm applicator seems reasonable.

Overall, our SRT‐100 Vision unit has demonstrated mechanical robustness. Two knobs of treatment arms, which are fastened after positioning of the applicator on the skin, became permanently loose when the treatment arms were often stretched without the knobs being completely unscrewed. Consequently, the knobs had to be replaced twice. Since then, the knobs have been used with greater attention to thread position, and the x‐ray tube head sagging has been closely checked not to endanger patient. The SRT‐100 Vision unit does not have a tube head angle indicator and therefore, tube head angle readouts were not tested as part of mechanical QA.

The results for spatial integrity check on ultrasound images were acceptable. However, the maximum deviation of 0.4 mm for the length of 2.0 mm seems large. Our experience is that incomplete filling of the probe with distilled water can result in inaccurate image acquisition, leading to a larger deviation of measurement. As QA experience was gained, image acquisition and measurement were improved.

In our clinic, ultrasound is uncommonly used, and minimum QA tests (spatial integrity and image quality) have been performed. Because of the high frequency (20 MHz) ultrasound of the SRT‐100 Vision unit, a penetration depth is about 5.0 mm. However, commercial ultrasound phantoms are typically constructed for lower frequency ultrasound (e.g., 8–12 MHz) and testing objects inside phantoms are located deeper (> 5.0 mm) and are not adequate for our ultrasound system. Designing and building our in‐house phantom to implement other QA tests such as grayscale visibility and axial and lateral resolutions recommended in AAPM TG 128^16^ will be warranted.

Annual radiation surveys including leakage radiation around the SRT‐100 Vision unit were not included in our QA program. Radiation surveys are typically conduced at the time of commissioning to confirm that a shielding design goal is met. However, annual radiation surveys are good clinical practice and need to be implemented in our QA program.

Unlike for other external beam radiation therapy units, an annual end to end test for 3D dosimetry using an in‐house or third‐party system was not performed. In SRT, a superficial lesion ≤ 5.0 mm in depth is treated and treatment time or monitor unit (MU) calculations are typically performed using absolute output (cGy/min or cGy/MU) and PDD measured in water assuming that underlining tissues are water equivalent. However, this assumption is not accurate due to various tissue types such as bone, air, adipose, and muscle underneath or near the lesion. To date, no United States Food and Drug Administration approved treatment planning system provides 3D dose distributions for SRT. Development of 3D dosimetry in SRT is of interest and will be warranted in the future.

## 5 CONCLUSIONS

The results of periodic machine QA tests encompassing dosimetry, mechanical, safety and imaging tests over the first three and a half years of operation were well within the recommended tolerance, thereby demonstrating high performance and stability of our SRT‐100 Vision unit.

## AUTHOR CONTRIBUTIONS

Yongsook C. Lee designed the concept, performed measurements, and wrote the initial version of the manuscript. William Romaguera performed measurements. Stephen D. Davis, D Jay Wieczorek, Vibha Chaswal, Ranjini Tolakanahalli, Minesh P. Mehta, Noah S. Kalman, and Alonso N. Gutierrez provided scientific reviews and contributed to the final manuscript.

## CONFLICT OF INTEREST STATEMENT

The authors declare no conflicts of interest.

## Supporting information



Supporting Information

## Data Availability

Data are not shared.
