# Supplementary material for: Initial three‐ and half‐year machine performance assessment of a superficial radiation therapy unit
Source: J Appl Clin Med Phys. 2025 Sep 21;26(10):e70258. doi: 10.1002/acm2.70258 (PMC12450352; doi:10.1002/acm2.70258)
Supplement: Supplementary file 1 — Supporting Information [file ACM2-26-e70258-s001.docx]

**Supplementary material**

**Table A** Mean and standard deviation (SD) of applicator factors measured each year

| **50** **kV_p_** |  | **1.5 cm** | **2.0 cm** | **2.5 cm** | **3.0 cm** | **4.0 cm** | **5.0 cm** |
| --- | --- | --- | --- | --- | --- | --- | --- |
| **2021** | **Mean** | 0.972 | 0.975 | 0.987 | 0.993 | 0.998 | 1.000 |
|  | **SD** | 0.001 | 0.000 | 0.000 | 0.000 | 0.000 | 0.000 |
| **2022** | **Mean** | 0.970 | 0.974 | 0.986 | 0.992 | 0.997 | 1.000 |
|  | **SD** | 0.001 | 0.000 | 0.001 | 0.001 | 0.000 | 0.000 |
| **2023** | **Mean** | 0.972 | 0.976 | 0.986 | 0.993 | 0.997 | 1.000 |
|  | **SD** | 0.000 | 0.001 | 0.000 | 0.000 | 0.000 | 0.000 |
| **2024** | **Mean** | 0.972 | 0.975 | 0.986 | 0.993 | 0.997 | 1.000 |
|  | **SD** | 0.000 | 0.000 | 0.000 | 0.000 | 0.000 | 0.000 |
| **70 kV_p_** |  | **1.5 cm** | **2.0 cm** | **2.5 cm** | **3.0 cm** | **4.0 cm** | **5.0 cm** |
| **2021** | **Mean** | 0.963 | 0.967 | 0.983 | 0.990 | 0.997 | 1.000 |
|  | **SD** | 0.001 | 0.000 | 0.000 | 0.001 | 0.000 | 0.000 |
| **2022** | **Mean** | 0.962 | 0.967 | 0.982 | 0.990 | 0.996 | 1.000 |
|  | **SD** | 0.000 | 0.000 | 0.001 | 0.000 | 0.000 | 0.000 |
| **2023** | **Mean** | 0.963 | 0.968 | 0.982 | 0.990 | 0.997 | 1.000 |
|  | **SD** | 0.000 | 0.001 | 0.001 | 0.001 | 0.001 | 0.000 |
| **2024** | **Mean** | 0.963 | 0.967 | 0.981 | 0.990 | 0.997 | 1.000 |
|  | **SD** | 0.000 | 0.001 | 0.000 | 0.000 | 0.001 | 0.000 |
| **100 kV_p_** |  | **1.5 cm** | **2.0 cm** | **2.5 cm** | **3.0 cm** | **4.0 cm** | **5.0 cm** |
| **2021** | **Mean** | 0.959 | 0.963 | 0.981 | 0.991 | 0.996 | 1.000 |
|  | **SD** | 0.001 | 0.000 | 0.000 | 0.000 | 0.000 | 0.000 |
| **2022** | **Mean** | 0.957 | 0.963 | 0.980 | 0.990 | 0.997 | 1.000 |
|  | **SD** | 0.001 | 0.001 | 0.000 | 0.001 | 0.001 | 0.000 |
| **2023** | **Mean** | 0.957 | 0.963 | 0.980 | 0.990 | 0.996 | 1.000 |
|  | **SD** | 0.001 | 0.001 | 0.001 | 0.001 | 0.001 | 0.000 |
| **2024** | **Mean** | 0.957 | 0.963 | 0.979 | 0.989 | 0.996 | 1.000 |
|  | **SD** | 0.001 | 0.001 | 0.001 | 0.001 | 0.001 | 0.000 |

**Table B** Mean and standard deviation (SD) of absolute outputs (absorbed dose to water on the water surface [cGy/min]) measured each year

| **50 kV_p_** |  | **1.5 cm** | **2.0 cm** | **2.5 cm** | **3.0 cm** | **4.0 cm** | **5.0 cm** |
| --- | --- | --- | --- | --- | --- | --- | --- |
| **2021** | **Mean** | 755.8 | 767.3 | 779.5 | 787.9 | 795.8 | 802.0 |
|  | **SD** | 0.04 | 0.04 | 0.04 | 0.04 | 0.04 | 0.04 |
| **2022** | **Mean** | 755.5 | 766.5 | 778.9 | 787.4 | 795.0 | 801.2 |
|  | **SD** | 0.18 | 0.18 | 0.18 | 0.19 | 0.19 | 0.19 |
| **2023** | **Mean** | 761.9 | 772.8 | 785.9 | 794.2 | 801.9 | 808.0 |
|  | **SD** | 0.52 | 0.52 | 0.53 | 0.54 | 0.54 | 0.55 |
| **2024** | **Mean** | 764.5 | 775.4 | 788.5 | 796.9 | 804.6 | 810.7 |
|  | **SD** | 0.15 | 0.16 | 0.16 | 0.16 | 0.16 | 0.16 |
| **70 kV_p_** |  | **1.5 cm** | **2.0 cm** | **2.5 cm** | **3.0 cm** | **4.0 cm** | **5.0 cm** |
| **2021** | **Mean** | 624.7 | 638.9 | 655.5 | 667.1 | 679.1 | 690.2 |
|  | **SD** | 0.28 | 0.29 | 0.30 | 0.30 | 0.31 | 0.31 |
| **2022** | **Mean** | 623.1 | 637.5 | 653.9 | 665.3 | 677.2 | 688.4 |
|  | **SD** | 0.19 | 0.19 | 0.20 | 0.20 | 0.20 | 0.21 |
| **2023** | **Mean** | 625.1 | 639.5 | 655.9 | 667.7 | 679.6 | 690.2 |
|  | **SD** | 0.50 | 0.51 | 0.53 | 0.54 | 0.55 | 0.56 |
| **2024** | **Mean** | 625.9 | 640.3 | 656.7 | 668.5 | 680.4 | 691.1 |
|  | **SD** | 0.50 | 0.51 | 0.52 | 0.53 | 0.54 | 0.55 |
| **100 kV_p_** |  | **1.5 cm** | **2.0 cm** | **2.5 cm** | **3.0 cm** | **4.0 cm** | **5.0 cm** |
| **2021** | **Mean** | 608.9 | 626.5 | 646.4 | 662.0 | 678.5 | 693.2 |
|  | **SD** | 0.27 | 0.28 | 0.29 | 0.29 | 0.30 | 0.31 |
| **2022** | **Mean** | 605.8 | 623.7 | 643.3 | 658.6 | 675.5 | 689.9 |
|  | **SD** | 0.25 | 0.25 | 0.26 | 0.27 | 0.28 | 0.28 |
| **2023** | **Mean** | 607.6 | 625.3 | 644.9 | 660.2 | 677.3 | 691.7 |
|  | **SD** | 0.28 | 0.29 | 0.30 | 0.30 | 0.31 | 0.32 |
| **2024** | **Mean** | 604.0 | 621.6 | 641.1 | 656.3 | 673.2 | 687.6 |
|  | **SD** | 0.13 | 0.13 | 0.14 | 0.14 | 0.14 | 0.15 |

**Table C** Mean and standard deviation (SD) of charges (pC) at each timer setting for output linearity measured each year

| **50 kV_p_** |  | **0.1 min** | **0.2 min** | **0.4 min** | **0.6 min** | **0.8 min** | **1.0 min** |
| --- | --- | --- | --- | --- | --- | --- | --- |
| **2021** | **Mean** | 117.3 | 234.5 | 468.8 | 703.2 | 937.7 | 1171.4 |
|  | **SD** | 0.03 | 0.20 | 0.12 | 0.31 | 0.06 | 1.27 |
| **2022** | **Mean** | 116.5 | 233.0 | 465.7 | 698.5 | 932.0 | 1164.6 |
|  | **SD** | 0.01 | 0.04 | 0.23 | 0.03 | 0.21 | 0.71 |
| **2023** | **Mean** | 115.0 | 229.8 | 459.8 | 690.2 | 920.8 | 1150.8 |
|  | **SD** | 0.12 | 0.13 | 0.24 | 0.70 | 0.76 | 0.21 |
| **2024** | **Mean** | 116.0 | 231.8 | 463.3 | 695.2 | 926.7 | 1158.6 |
|  | **SD** | 0.08 | 0.13 | 0.24 | 0.29 | 1.05 | 0.56 |
| **70 kV_p_** |  | **0.1 min** | **0.2 min** | **0.4 min** | **0.6 min** | **0.8 min** | **1.0 min** |
| **2021** | **Mean** | 110.1 | 220.1 | 440.4 | 661.0 | 881.3 | 1101.4 |
|  | **SD** | 0.16 | 0.20 | 0.13 | 0.36 | 0.68 | 0.61 |
| **2022** | **Mean** | 109.2 | 218.3 | 436.1 | 654.2 | 872.5 | 1090.5 |
|  | **SD** | 0.06 | 0.04 | 0.06 | 0.01 | 0.25 | 0.57 |
| **2023** | **Mean** | 107.8 | 215.8 | 431.6 | 646.9 | 863.0 | 1078.8 |
|  | **SD** | 0.14 | 0.03 | 0.36 | 0.15 | 0.75 | 1.42 |
| **2024** | **Mean** | 108.7 | 217.1 | 434.2 | 651.2 | 868.3 | 1086.0 |
|  | **SD** | 0.08 | 0.11 | 0.03 | 0.31 | 0.19 | 0.26 |
| **100 kV_p_** |  | **0.1 min** | **0.2 min** | **0.4 min** | **0.6 min** | **0.8 min** | **1.0 min** |
| **2021** | **Mean** | 109.3 | 218.3 | 436.7 | 654.8 | 872.5 | 1089.8 |
|  | **SD** | 0.04 | 0.14 | 0.22 | 0.13 | 0.34 | 0.95 |
| **2022** | **Mean** | 108.3 | 216.3 | 432.6 | 649.1 | 866.1 | 1082.3 |
|  | **SD** | 0.01 | 0.04 | 0.13 | 0.18 | 0.16 | 0.14 |
| **2023** | **Mean** | 106.3 | 212.6 | 425.0 | 637.8 | 850.4 | 1063.1 |
|  | **SD** | 0.14 | 0.09 | 0.06 | 0.08 | 0.10 | 1.10 |
| **2024** | **Mean** | 107.0 | 213.8 | 427.4 | 641.2 | 854.3 | 1066.9 |
|  | **SD** | 0.13 | 0.03 | 0.09 | 0.35 | 0.54 | 0.93 |

**Table D** Mean and standard deviation (SD) of percent depth dose (%) measured each year

| **50 kV_p_** |  | **0.0 mm** | **1.0 mm** | **3.0 mm** | **5.0 mm** |
| --- | --- | --- | --- | --- | --- |
| **2021** | **Mean** | 100.0 | 82.9 | 60.4 | 47.0 |
|  | **SD** | 0.00 | 0.01 | 0.02 | 0.01 |
| **2022** | **Mean** | 100.0 | 82.9 | 58.9 | 47.1 |
|  | **SD** | 0.00 | 0.01 | 0.01 | 0.01 |
| **2023** | **Mean** | 100.0 | 83.0 | 58.3 | 46.5 |
|  | **SD** | 0.00 | 0.03 | 0.01 | 0.01 |
| **2024** | **Mean** | 100.0 | 83.1 | 58.8 | 47.0 |
|  | **SD** | 0.00 | 0.06 | 0.02 | 0.03 |
| **70 kV_p_** |  | **0.0 mm** | **1.0 mm** | **3.0 mm** | **5.0 mm** |
| **2021** | **Mean** | 100.0 | 90.5 | 77.3 | 67.2 |
|  | **SD** | 0.00 | 0.02 | 0.01 | 0.02 |
| **2022** | **Mean** | 100.0 | 90.7 | 76.4 | 67.1 |
|  | **SD** | 0.00 | 0.02 | 0.00 | 0.04 |
| **2023** | **Mean** | 100.0 | 90.6 | 75.6 | 66.5 |
|  | **SD** | 0.00 | 0.03 | 0.01 | 0.02 |
| **2024** | **Mean** | 100.0 | 90.7 | 76.2 | 67.2 |
|  | **SD** | 0.00 | 0.01 | 0.05 | 0.00 |
| **100 kV_p_** |  | **0.0 mm** | **1.0 mm** | **3.0 mm** | **5.0 mm** |
| **2021** | **Mean** | 100.0 | 93.3 | 84.2 | 76.8 |
|  | **SD** | 0.00 | 0.04 | 0.02 | 0.00 |
| **2022** | **Mean** | 100.0 | 93.5 | 83.6 | 76.5 |
|  | **SD** | 0.00 | 0.04 | 0.06 | 0.04 |
| **2023** | **Mean** | 100.0 | 93.2 | 82.8 | 76.1 |
|  | **SD** | 0.00 | 0.03 | 0.05 | 0.01 |
| **2024** | **Mean** | 100.0 | 93.4 | 83.3 | 76.8 |
|  | **SD** | 0.00 | 0.03 | 0.04 | 0.02 |
